# High capacity reversible data hiding with interpolation and adaptive embedding

**DOI:** 10.1371/journal.pone.0212093

**Published:** 2019-03-06

**Authors:** Md. Abdul Wahed, Hussain Nyeem

**Affiliations:** Department of Electrical, Electronic and Communication Engineering (EECE) Military Institute of Science and Technology (MIST), Mirpur Cantonment, Dhaka-1216; Mar Ephraem College of Engineering & Technology, INDIA

## Abstract

A new Interpolation based Reversible Data Hiding (IRDH) scheme is reported in this paper. For different applications of an IRDH scheme to the digital image, video, multimedia, big-data and biological data, the embedding capacity requirement usually varies. Disregarding this important consideration, existing IRDH schemes do not offer a better embedding rate-distortion performance for varying size payloads. To attain this varying capacity requirement with our proposed adaptive embedding, we formulate a capacity control parameter and propose to utilize it to determine a minimum set of embeddable bits in a pixel. Additionally, we use a logical (or bit-wise) correlation between the embeddable pixel and estimated versions of an embedded pixel. Thereby, while a higher range between an upper and lower limit of the embedding capacity is maintained, a given capacity requirement within that limit is also attained with a better-embedded image quality. Computational modeling of all new processes of the scheme is presented, and performance of the scheme is evaluated with a set of popular test-images. Experimental results of our proposed scheme compared to the prominent IRDH schemes have recorded a significantly better-embedding rate-distortion performance.

## Introduction

Reversible data hiding (RDH), also known as a reversible or lossless watermarking, is being widely investigated for different applications to the digital image, video, multimedia, big-data and biological data [1–10]. An RDH scheme generally embeds *payload* (*i.e*., secret data with any side information) in a cover image (or other data like audio, video or DNA) such that the embedded payload can completely be extracted followed by an exact recovery of the input image [9, 10]. An RDH scheme usually aims to attain better (embedding) *rate-distortion* performance. Thus, for the higher embedding capacity (or rate) with a lower embedding distortion, different RDH schemes have been developed, for example, *DLE*–Direct Least-significant-bit (LSB) Embedding–schemes [11–15], Difference Expansion (DE) schemes [16, 17], Histogram Shifting (HS) schemes [18, 19], Reversible Contrast Matching (RCM) schemes [20], Prediction Error Expansion (PEE) schemes [21, 22].

Recently, interpolation-based RDH (IRDH) schemes are being studied for a better rate-distortion performance [13–15, 23–27]. We will briefly review those schemes in Section State of the IRDH schemes showing that their development mainly focuses on the improvement of two underlying processes: (*i*) computation of interpolated pixels and (*ii*) embedding of payload. For computing interpolated pixels, the parabolic interpolation (PI) offers better image quality so far [13–15]. For embedding, interpolated pixels of an input image are generally used, keeping the original pixels untouched, which is desirable in the military and medical image applications.

Additionally, while a better visual quality of the embedded images can be obtained with the existing IRDH schemes, a varying embedding capacity requirement (*i.e*., varying size payloads) is yet to be considered. For different multi-disciplinary applications of an RDH scheme, the payload size usually varies. It is generally assumed that embedding will continue for the last bit of payload within the capacity limit that may result in non-uniform distortion in the embedded image [28]. Thus the best possible image quality for embedding varying size payloads cannot always be ensured. This embedding problem of varying size payload can be addressed using adaptive embedding, which again poses three main challenges: (*i*) defining a universal capacity control parameter to deterministically allocate room for payload bits, (*ii*) keeping dynamic range of the embedding capacity possibly ‘higher’ to attain the varying capacity requirement of a target application, and (*iii*) maintaining a uniform distribution of the embeddable bits for minimum possible distortion resulting in a better rate-distortion performance.

As a first step to tackle the above challenges, we employed a capacity control parameter in [14, 15] that deterministically selects a set of bit-planes for embedding original or complement form of payload bits. For tracking these versions of the embedded data (*i.e*., original or complement), a flag bit was introduced. The PI technique in [13] was simplified for efficiently computing an interpolated image in [14, 15]. While a high dynamic range of embedding capacity was obtained with our initially developed schemes [14, 15], the capacity can be further increased if the use of flag bits can be avoided. It means, one more payload-bit can be embedded replacing a flag bit in a pixel with improved embedded image quality.

Therefore, as a primary contribution of this paper (see Section Scope of improvement for more details), we present a new adaptive IRDH scheme. We use the simplified PI technique (SPI) [15] for up-sampling an input image (Section Image up-sampling). An adaptive embedding with a capacity control parameter is more formally defined and employed for embedding of varying size payloads (Section Proposed adaptive embedding). We utilize a logical (or bit-wise) correlation between the embeddable pixel and estimated versions of an embedded pixel to avoid the use of any flag bit. This scheme is thus envisaged to have a significantly better-embedding rate-distortion performance (Section Results and analysis) and would create a new paradigm in adaptive IRDH research for different multi-disciplinary applications.

## State of the IRDH schemes

**Fig 1:**
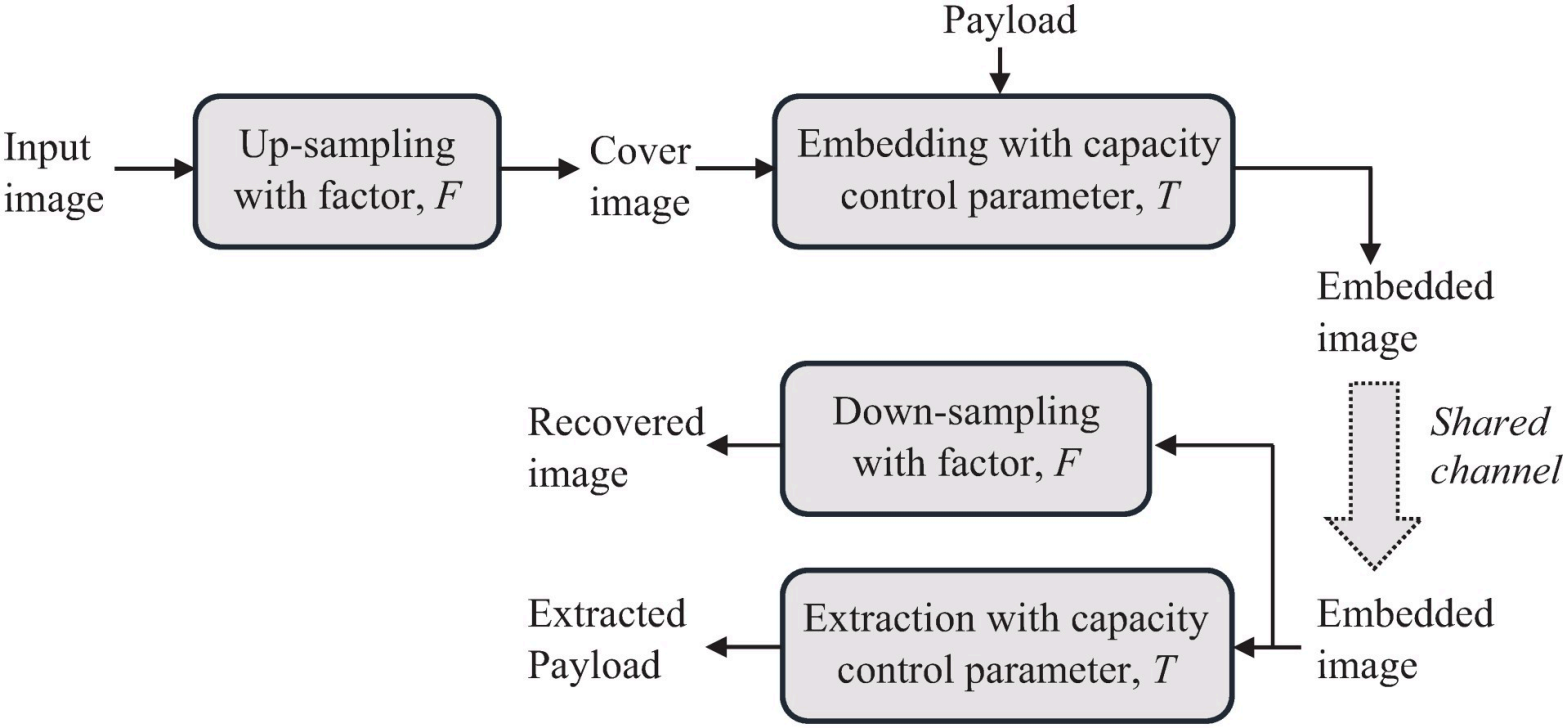
A general framework of the proposed IRDH scheme.

In this section, we review the popular interpolation techniques and their uses in IRDH schemes. Interpolation techniques are chosen to obtain an up-sampled image containing both the original and interpolated pixels. Since the image quality of the up-sampled image eventually contributes to maintaining a better-embedded image quality, lower distortion in the up-sampled image is always desirable. We note that for evaluating distortion between an original image and its interpolated version, the original image is first down-sampled followed by the up-sampling using the interpolation technique in question. Unlike this, in an IRDH scheme, an input image is directly up-sampled (using interpolation) for embedding (see Section Modeling our proposed IRDH scheme and Fig 1), where interpolated pixels are used in different ways to achieve a better rate-distortion performance, which is discussed in the sections below.

### Interpolation techniques

Different interpolation techniques have been employed for up-sampling the cover image during embedding. For example, neighbor mean interpolation (NMI) [23], interpolation by neighboring pixels (INP) [25], enhanced neighbor mean interpolation (ENMI) [27] and parabolic interpolation (PI) [13] are a few that showed great promises for IRDH schemes. Additionally, bilinear interpolation (BI) and nearest neighbor interpolation (NNI) are two basic interpolation techniques that have not been employed in IRDH schemes, but they are used to compare the performance of interpolation in IRDH schemes (for example, see [13, 29]).

The NMI technique [23] computes an interpolated pixel as the average of the nearest original pixels. For the horizontal or vertical neighborhood, two nearest pixels are considered and for the diagonal neighborhood, three nearest pixels are considered. Thereby, a 2 × 2 block of an input image is up-sampled to a size of 3 × 3 containing five interpolated pixels. While this technique offers reasonably good up-sampled image quality, a better quality up-sampled image can be obtained by redefining the neighborhood and interpolated pixels for computing the final interpolated pixels as reported in IRDH schemes with INP [25] and ENMI [27].

However, for further improvement of the interpolated image quality, PI has later been introduced in an IRDH scheme [13]. With a set of three known pixels (*i.e*., original pixels), two unknown pixels are computed as such an original image block of size 1 × 3 is up-sampled to a block of size 1 × 5. To obtain an up-sampled pixel a weighted average of its all possible interpolated values and other nearest neighborhood pixels (that are not considered for interpolation) is used. This computation continues for all overlapping blocks of original pixels with separate consideration of interpolated pixels in the image border. With a relatively higher computational complexity, this technique produces the best quality interpolated image so far.

We have therefore simplified the PI technique of Zhang *et al*. [14] using only non-overlapping image blocks. To avoid the inter-block visual artifact, we also improved that simplified technique later in [15] with overlapping blocks, where an interpolated pixel in a block is computed once in each direction (*i.e*., horizontal, vertical and diagonal) (see Section Modeling our proposed IRDH scheme). Whereas, in Zhang *et al*.’s PI technique, as mentioned above, multiple versions of each interpolated pixel are computed to get a closer estimate. Thus, our simplified PI [15] (that we call SPI technique in what follows) significantly reduces the computational complexity of Zhang *et al*.’s PI technique to obtain similar embedded image quality. This will be demonstrated in terms of run-time and embedded image quality with some new results in Section Results and analysis. A minimal working example of our SPI technique is illustrated in Section A working example.

### Embedding techniques based on interpolation

We now briefly discuss the use of interpolation techniques in IRDH schemes. The main uses of interpolation are found for (*i*) computing prediction errors (PEE-based schemes [30–32]), (*ii*) computing embeddable bits (DLE-based schemes [23–25]), and (*iii*) a combination of both [27]. An IRDH scheme with PEE computes the predicted errors from the cover image and interpolated image, and the secret data are embedded by modifying the predicted errors in a subset of original pixels. Here, reversibility of these schemes depends on the PEE process. In contrast, an IRDH scheme with DLE embeds secret data only in the interpolated pixels by replacing their LSBs, keeping the original pixels untouched. This embedding is particularly useful in applications like medical and military imaging, where minimum changes in the cover images (*i.e*., original pixels) are usually restricted [29, 33–37].

The DLE-based schemes are relatively simple and offer better user access control than the other IRDH schemes mentioned above. This means that while the secret data may be extracted by an authorized user (*i.e*., who has the privilege to obtain the embedded data), the cover image may be independently restored by any user who wants to see it original. For this purpose, a suitable cryptographic tool with private or public keys may be employed for encrypting payload, which is beyond the scope of this paper. In summary, IRDH schemes with DLE have several advantages over its counterpart as noted below:

- embedding and extraction processes are relatively simple
- a location map is not usually required
- embedded image being up-sampled provides a higher spatial resolution
- the original pixels remain untouched (required in some military and medical applications)
- instant recovery of the cover image (with or without extraction of the embedded data) by down-sampling
- a better user access control to the embedded data and cover image, and
- a relatively higher embedding capacity with reasonably better image quality.

Although a combination of the DLE and PEE techniques in IRDH scheme has also been reported in [27] to offer a better image quality and higher embedding capacity, the above advantage of the DLE-based schemes may no longer exist. That type of IRDH scheme thus can be *semi-reversible* (*i.e*., the embedded data can be completely extracted, while the original image can be partially recovered), and the original pixels cannot also be preserved intact in the embedded image. In this paper, we, therefore, limit our focus to the development of a DLE-based IRDH scheme.

A recent DLE based IRDH scheme reported in [29] introduced Base-3 conversion of the embeddable data bits and demonstrated an improved rate-distortion performance with the embedding of a large size payload, which includes location maps for complete extraction of the embedded data. Although those location maps are compressed and embedded along with the base-converted secret data, this essentially reduces the effective embedding capacity of the scheme [38]. This also means that with any suitable compression and/or base-conversion of payloads may also improve the embedding capacity of other existing IRDH schemes including our scheme presented in this paper, which is left beyond the scope of this paper and mentioned in Section Conclusion.

Other prominent DLE-based IRDH schemes [13, 23, 25] also improved interpolated image quality for high embedding capacity requiring no location map and compression technique. However, they do not account for any varying capacity requirement, which leaves a room for improvement of the IRDH scheme for embedding varying size payload. Therefore, in this paper, we develop and present a new IRDH scheme for high capacity adaptive data hiding. Technical details of the proposed scheme are discussed in Section Modeling our proposed IRDH scheme for the envisaged improvements noted in the section below.

### Scope of improvement

The current state of the IRDH schemes, as discussed above, demands a suitable adaptive embedding process that is computationally efficient and offers a better rate-distortion performance, particularly for varying embedding capacity requirements. Our research presented in this paper thus contributes to the development of an adaptive IRDH scheme as follows.

- An SPI technique is developed for efficient up-sampling of the input image.
- A capacity control parameter is formulated and employed in developing a new adaptive embedding process to deterministically allocate the room for embedding.
- A better-embedded image quality is ensured with a closer estimate of the embeddable pixel.
- Computational models of all key processes of the proposed IRDH scheme are developed.
- Rate-distortion performance of the proposed IRDH scheme is evaluated, analyzed and validated with the prominent IRDH schemes [13, 23, 25] and our earlier scheme [15].

The early result of our work with the SPI technique has been presented in [14, 15], as discussed in Section Image up-sampling. For embedding, we employed a capacity control parameter in [14, 15] to allocate the room for embedding. To minimize embedding distortion, we computed a closer estimate of the embeddable pixels using either the original or the complement of the payload bits. To track the versions of the embedded bits, we used a flag bit. However, the embedding capacity of those schemes can further be improved if an additional bit can be embedded in place of the flag bit.

In this paper, we investigate for the use of a logical (*i.e*. bit-wise) correlation between the embeddable pixels and two versions (*i.e*., original and complement) of embedded pixels. Thereby, the use of flag-bit can be avoided to embed one more payload bit in every place of flag-bits, which would significantly increase the embedding capacity with no additional embedding distortion (see Section Modeling our proposed IRDH scheme). We thus develop a new adaptive IRDH scheme for varying size payload embedding with the best possible embedded image quality. We formulate the capacity control parameter more formally and present the simplified up-sampling, new embedding and extraction processes with their algorithmic details in Section Modeling our proposed IRDH scheme. Moreover, we analyze the performance of our schemes with new results by comparing it with the IRDH schemes in [13, 15, 23, 25] (see Section Results and analysis).

## Modeling our proposed IRDH scheme

A general framework of our proposed IRDH scheme is presented in Fig 1. Three main processes of the scheme (*i.e*., image up-sampling, embedding, and extraction) are modeled and discussed in this section below. We adopt the general notation of the data hiding framework from [10, 39], which is summarized in Table 1. We also differentiate between the embeddable and interpolated pixels as such the embeddable pixels are a sub-set of interpolated pixels, where the payload is embedded in. The other interpolated pixels (*i.e*., the last three interpolated pixels, see the last paragraph of Section Proposed adaptive embedding) are used to carry any side information required to execute the extraction and recovery processes.

**Table 1:** Key nomenclature for our proposed IRDH framework.

Notation	Term
*I*	input image of size *M* × *N* × *L* (*i.e*., a set of *M* × *N* pixels)
*M*	number of pixels in a row of *I*: M∈ℕ
*N*	number of pixels in a column of *I*: N∈ℕ
*L*	bit-depth of an image, L∈ℕ
*F*	up-sampling factor, F∈ℕ
*I*_*up*_	initialized up-sampled image of size (*FM* − 1) × (*FN* − 1) × *L*, *i.e*., *I*_*up*_ − *I* = {0}
$I_{up}^{\prime }$	up-sampled image of size (*FM* − 1) × (*FN* − 1) × *L* with interpolated pixels
*C*	interpolated or cover image of size *FM* × *FN* × *L*
*I*_*em*_	embedded image of size *FM* × *FN* × *L*
*y*	an image pixel
*n*	bit-length of *y* as in Eq (5)
*n*_*em*_	number of embeddable bits of *y* as in Eq (6)
*y*_*i*_, *y*_*i*,*j*_	*y* of *i*^*th*^ or (*i*, *j*)^*th*^ position in 1D or 2D array, respectively, which also applies to *y*′
*y*′	an embedded pixel
*data*	set of payload bits, {0, 1}^*γ*^
*γ*	embedding capacity requirement (*i.e*., size of payload in bits, *γ* = ||*data*||)
*T*	capacity control parameter (*i.e*., number of unchanged MSBs in *y* ∈ *C* − *I*)
$I_{Hblock},I_{Hblock}^{\prime }$	1 × 5 size image block of horizontal pixels: *I*_*Hblock*_ ⊂ *I*_*up*_ and $I_{Hblock}^{\prime }\subset I_{up}^{\prime }$
$I_{Vblock},I_{Vblock}^{\prime }$	1 × 5 size image block of vertical pixels: *I*_*Vblock*_ ⊂ *I*_*up*_ and $I_{Vblock}^{\prime }\subset I_{up}^{\prime }$
$I_{Dblock},I_{Dblock}^{\prime }$	1 × 5 size image block of diagonal pixels: *I*_*Dblock*_ ⊂ *I*_*up*_ and $I_{Dblock}^{\prime }\subset I_{up}^{\prime }$
*E*_*c*_	total embedding capacity in bit

### Image up-sampling

**Fig 2:**
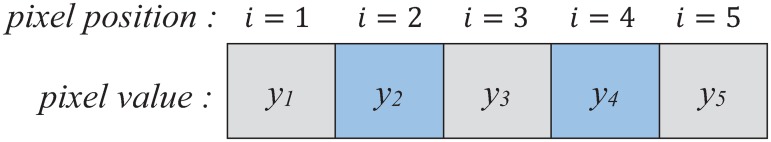
Pixel arrangement for a parabolic interpolation (PI).

We present here a general framework of PI first followed by its use in modeling SPI technique for up-sampling an input image. Let us consider a set of 5 consecutive pixels, {*y*_*i*_}: *y*_*i*_ ∈ {0, 2^*L*^ − 1} with respective pixel positions, *i* ∈ {1, 2, ⋯, 5}, where *L* is the bit-depth of the given image. This is illustrated in Fig 2. With this setting, the known pixels are {*y*_*i*_}: *i* ∈ {1, 3, 5} and the other two are the up-sampled pixels, *i.e*., {*y*_*i*_}: *i* ∈ {2, 4} that are unknown and will be computed using PI.

$$\begin{array}{c} \begin{array}{c} a.1^{2}+b.1+c=y_{1}\\ a.3^{2}+b.3+c=y_{3}\\ a.5^{2}+b.5+c=y_{5} \end{array}\} \end{array}$$ (1)

$$\begin{array}{c} \begin{array}{c} y_{2}=a.2^{2}+b.2+c\\ y_{4}=a.4^{2}+b.4+c \end{array}\} \end{array}$$ (2)

For computing the unknown pixels, *y*_2_ and *y*_4_, a set of three parabolic equations of the form *a*.*i*^2^ + *b*.*i* + *c* = *y*_*i*_ are obtained with the three known pixels as in Eq (1). The coefficients, *a*, *b*, and *c* are then obtained from the solution of these equations, which are used to compute the *y*_2_ and *y*_4_ as in Eq (2). This computation will be applied in each directional block (*i.e*., horizontal, vertical and diagonal) and is repeated for all blocks to compute an interpolated image. This SPI-based up-sampling is modeled with the function interp(⋅) in Algorithm 1.

**Algorithm 1**. interp (*I*, *F*)

**Input(s):** *I*, *F*

**Output(s):** *C*

**Begin**

1 (*M*, *N*) ← size(*I*)              ⊳ number of rows and columns of *I*

2 *I*_*up*_ ← upsample(*I*, *M*, *N*, *F*)       ⊳ up-sampling *I* to *I*_*up*_ by factor *F* to a size *FM* − 1 × *FN* − 1

3 {*I*_*Hblock*_} ← hbolck(*I*_*up*_)    ⊳ divides *I*_*up*_ into a set of overlapping horizontal blocks of size 1 × 5

4 {*I*_*Vblock*_} ← vbolck(*I*_*up*_) ⊳ divides *I*_*up*_ into a set of overlapping vertical blocks of size 1 × 5

5 {*I*_*Dblock*_} ← dbolck(*I*_*up*_) ⊳ divides *I*_*up*_ into a set of overlapping diagonal blocks of size 1 × 5

6 **for all** *I*_*Hblock*_ ⊂ *I*_*up*_ **do**

7  $I_{Hblock}^{\prime }\leftarrow \text{parabolic}\left(I_{Hblock}\right)$     ⊳ computes interpolated pixels in *I*_*Hblock*_

8 **end for**

9 **for all** *I*_*Vblock*_ ⊂ *I*_*up*_ **do**

10  $I_{Vblock}^{\prime }\leftarrow \text{parabolic}\left(I_{Vblock}\right)$     ⊳ computes interpolated pixels in *I*_*Vblock*_

11 **end for**

12 **for all** *I*_*Dblock*_ ⊂ *I*_*up*_ **do**

13  $I_{Dblock}^{\prime }\leftarrow \text{parabolic}\left(I_{Dblock}\right)$     ⊳ computes interpolated pixel sin *I*_*Dblock*_

14 **end for**

15 $I_{up}^{\prime }\leftarrow \text{construct}\left(\{I_{Hblock}^{\prime }\},\{I_{Vblock}^{\prime }\},\{I_{Dblock}^{\prime }\}\right)$   ⊳ updates *I*_*up*_ with $I_{Hblock}^{\prime }$, $I_{Vblock}^{\prime }$ and $I_{Dblock}^{\prime }$

16 $C\leftarrow \text{pad}\left(I_{up}^{\prime }\right)$ ⊳ padding in the last row and last column of $I_{up}^{\prime }$ to make its size *FM* × *FN*

17 Return *C*

**End**

**Fig 3:**
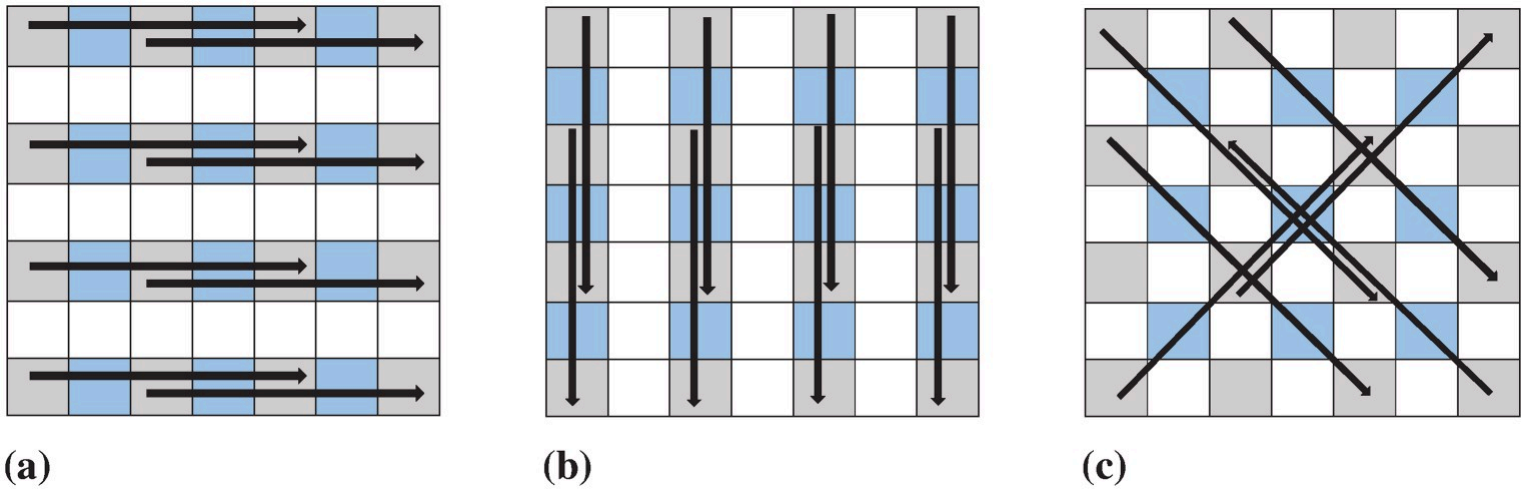
An example of directions in a block for SPI-based image up-sampling [15]: (a) horizontal, (b) vertical and (c) diagonal.

Particularly, interp(⋅) takes the original image, *I* and the factor, *F* as input to output an interpolated image, *C*. An up-sampled image is initialized with upsample(⋅) that inserts interleaving zero columns and zero rows in *I*. Thus, an *FM* − 1 × *FN* − 1 sized image, *I*_*up*_ is computed with the *M* × *N* sized *I*, which is later divided into a set of overlapping blocks of size 1 × 5 by scanning pixels in horizontal, vertical and diagonal directions as showed in Fig 3. Thus, as in Step 3-5 of the algorithm, {*I*_*Hblock*_}, {*I*_*Vblock*_} and {*I*_*Dblock*_} are the sets of horizontal, vertical and diagonal blocks obtained from the functions, hblock(⋅), vblock(⋅) and dblock(⋅), respectively.

Each directional block contains five consecutive pixels, where the 1*st*, 3*rd* and 5*th* pixels are the original, and the 2*nd* and 4*th* pixels are the newly inserted zero pixels, whose values are to be computed using Eqs (1) and (2) as defined in Steps 9-14 of Algorithm 1 with parabolic(⋅). Newly inserted zero pixel values of {*I*_*Hblock*_}, {*I*_*Vblock*_} and {*I*_*Dblock*_} are thus replaced with the interpolated values obtaining their respective interpolated blocks, {$I_{Hblock}^{\prime }$}, {$I_{Vblock}^{\prime }$} and {$I_{Dblock}^{\prime }$}. These interpolated blocks are then combined to construct $I_{up}^{\prime }$ using the function, construct(⋅). The last row and last column of $I_{up}^{\prime }$ is replicated as padded pixels to make the image size *FM* × *FN* using pad(⋅) in Step 16. For example, with *F* = 2, an input image, *I* of size *M* × *N* is finally up-sampled to an interpolated image, *C* of size 2*M* × 2*N*.

### Proposed adaptive embedding

We model here our proposed embedding such that it can adaptively embed the payload into the LSBs of the embeddable pixels. For this, we first formulate a capacity control parameter, *T* in Eq (3) for adaptive embedding, *i.e*., to attain any varying embedding capacity requirement.

$$\begin{array}{c} T\in \{t\}\subset N:1\leq t\leq (L-1)\;\text{and}\;L\;\text{is}\;\text{the}\;\text{bit-depth}\;\text{of}\;C \end{array}$$ (3)

**Algorithm 2**. embedding (*F*, *C*, *data*, *T*)

**Input(s):** *C*, *data*, *F*, *T*

**Output(s):** *I*_*em*_

**Begin**

1 **Initialize:**

 *startbit* ← 1

2 *I* ← downsample(*C*, *F*)            ⊳ down-sample *C* by *F*

3 **for all** *y* ∈ *C* − *I* **do**         ⊳ for all the embeddable pixels in *C*

4  *n* ← bitlength(*y*)        ⊳ returns bit-length of *y* using Eq (5)

5  **if** *n* ≤ 1 + *T* **then**

6   *n*_*em*_ ← 2       ⊳ *n*_*em*_ is the number of embeddable bits

7  **else**

8   *n*_*em*_ ← *n* − *T*

9  **end if**

10  *p* ← getLSB(*y*, *n*_*em*_)            ⊳ returns *n*_*em*_-LSBs of *y*

11  *d* ← getbit(*data*, *n*_*em*_, *startbit*)   ⊳ returns *n*_*em*_-bits of *data* from starting bit-position, *startbit*

12  $\bar{d}\leftarrow \text{complement}\left(d\right)$       ⊳ returns 2’s complement of *d*

13  *X*_*d*_ ← bitXOR(*p*, *d*)           ⊳ a bit wise XOR of *p* and *d*

14  $X_{\bar{d}}\leftarrow \text{bitXOR}\left(p,\bar{d}\right)$        ⊳ a bit wise XOR of *p* and $\bar{d}$

15  *y*_*d*_ ← substituteLSB(*y*, *d*)   ⊳ returns a version of embedded pixel substituting *d* for *n*_*em*_-LSBs of *y*

16  $y_{\bar{d}}\leftarrow \text{substituteLSB}\left(y,\bar{d}\right)$ ⊳ returns another version of embedded pixel substituting $\bar{d}$ for *n*_*em*_-LSBs of *y*

17  *startbit* ← *startbit* + *n*_*em*_

18  **if** $\left|X_{d}|\leq |X_{\bar{d}}\right|$ **then**

19   *y*′ ← *y*_*d*_

20  **else**

21   $y^{\prime }\leftarrow y_{\bar{d}}$

22  **end if**

23  *y* ← *y*′                 ⊳ updating *y* with *y*′

24 **end for**

25 Return *I*_*em*_

**End**

In general, *T* is a predefined number of unchanged MSBs. So, the lesser is the value of *T*, the greater are the number of embeddable LSBs, and so is the embedding capacity. However, with a higher capacity requirement, embedding distortion is usually higher. So, a maximum possible value of *T* is required as such the capacity requirement, *γ* is attained with the best possible embedded image quality. We model the process of computing *T* in Algorithm 3, where *T* lies in the range [1, *L* − 1]. For different values of *T*, the embedding capacity, *E*_*c*_ is computed in Step 10 of Algorithm 3 using Eq (4a), which is the total number of embeddable bits, *i.e*., ∑*n*_*em*_ for all *y* ∈ *C* − *I* as per Eq (6).

**Algorithm 3**. c.parameter (*C*, *data*, *F*)

**Input(s):** *C*, *data*, *F*

**Output(s):** *T*

**Begin**

1 **Initialize:**

  *T* ← (*L* − 1)

  *γ* ← size(*data*)           ⊳ *γ* is the size of payload, *data*

2 *I* ← downsample(*C*, *F*)          ⊳ down-sample *C* by *F*

3 *N*_1_ ← 0      ⊳ *N*_1_ is the total pixels with two embeddable LSBs as per Eq (6)

4 **for all** *y* ∈ *C* − *I* **do**         ⊳ for all embeddable pixels in *C*

5  *n* ← bitlength(*y*)      ⊳ returns bit-length of *y* using Eq (5)

6  **if** *n* ≤ 1 + *T* **then**

7   *N*_1_ ← (*N*_1_ + 1)

8  **end if**

9 **end for**

10 *E*_*c*_ ← *N*_1_ × 2 + {(*F* − 1)(*M* × *N*) − *N*_1_} × (*n* − *T*)    ⊳ *E*_*c*_ is total embedding capacity

11 **while** *E*_*c*_ ≤ *γ* **do**

12  **if** *T* ≥ 2 **then**

13   *T* ← (*T* − 1)

14   repeat step 3 to step 10        ⊳ recalculate *E*_*c*_ for new *T*

15  **else**

16   break and reconsider *F*         ⊳ Increase *F* to attain *γ*

17  **end if**

18 **end while**

19 Return *T*

**End**

Algorithm 3 illustrates that with the initialized *T* = *L* − 1 (*i.e*., the largest value of *T*), *E*_*c*_ is computed to verify if the embedding capacity requirement, *γ* is attained. If not, the value of *T* is further decreased and this process continues until Eq (4b) is satisfied. We note here that, with the lowest value of *T* (*i.e*., *T* = 1), the capacity condition in Eq (4b) may not be fulfilled for some large size payload, *γ*. This means that the payload size may sometimes exceed the maximum embedding capacity. While this condition may also be true for other RDH schemes, we leave an option to reconsider the up-sampling factor, *F* to increase the number of embeddable pixels. However, we here illustrate a case of our IRDH scheme with *F* = 2, which may be increased for a higher embedding capacity requirement with a higher size interpolated (or cover) image.

$$\begin{array}{c} E_{c}=N_{1}\times 2+\left\{\left(F-1\right)\left(M\times N\right)-N_{1}\right)\}\times \left(n-T\right) \end{array}$$ (4a)

$$\begin{array}{c} E_{c}\geq \gamma \end{array}$$ (4b)

Once *T* is obtained for a capacity requirement, *γ*, the embedding process is invoked for embedding given payload bits. The proposed embedding is modeled in Algorithm 2. The algorithm takes *C*, *data*, *F* and *T* as a set of inputs and returns the embedded image, *I*_*em*_. For example, with a given embeddable pixel *y* ∈ *C* − *I* the bit length, *n* is calculated by the function bitlength(⋅) using Eq (5). The number of embeddable LSBs, *n*_*em*_ is then computed according to the condition given in Eq (6) by the function getnem(⋅). Now, *n*_*em*_-number of LSBs of *y* is compared with the *n*_*em*_-bit *data*, *d* and its complement, $\bar{d}$ using a bit-wise XOR operation. Thus, their logical differences, *X*_*d*_ and $X_{\bar{d}}$ are computed, respectively. These *X*_*d*_ and $X_{\bar{d}}$ are compared according to the condition given in Eq (7) to choose the final embedded pixel, *y*′ either from *y*_*d*_ or $y_{\bar{d}}$. (Here, *y*_*d*_ and $y_{\bar{d}}$ are the two versions of embedded pixel computed using substituteLSB(⋅); the first version replaces the LSBs of *y* with the *d*, and the second one replaces the same with $\bar{d}$.) In other words, $\left|X_{d}|\leq \;|X_{\bar{d}}\right|$ means that embeddable pixel version, *y*_*d*_ is closer to *y*. So, the embedded pixel, *y*′ would take the value of *y*_*d*_; otherwise, the embedded pixel would be $y_{\bar{d}}$. Continuing this embedding for all embeddable pixels, an embedded image, *I*_*em*_ is obtained.

$$\begin{array}{c} n=\{\begin{array}{cc} \;\lceil \log_{2}(y)\rceil & \;\text{if}\;y>1\\ \;1 & \;\text{otherwise}\; \end{array} \end{array}$$ (5)

$$\begin{array}{c} n_{em}=\{\begin{array}{cc} \;2 & \;\text{if}\;n\leq 1+T\\ \;n-T & \;\text{otherwise}\; \end{array} \end{array}$$ (6)

$$\begin{array}{c} y^{\prime }=\{\begin{array}{cc} \;y_{d} & \;\text{if}\;|X_{d}\left|\leq \right|X_{\bar{d}}|\\ \;y_{\bar{d}} & \;\text{otherwise} \end{array} \end{array}$$ (7)

For a blind operation of the recovery and extraction processes, the value of *F* and *T* are to be stored as side information in the *I*_*em*_ such that the cover image can completely be recovered and the embedded data are exactly extracted from *I*_*em*_. For this, the last three interpolated pixels are excluded for embedding. In each of these pixels, two bits of the 6-bit side information (the first 3-bits are for *F* and the rest 3-bits are for *T*) are embedded so that the receiver can extract these bits to execute the extraction and recovery processes. The other interpolated pixels used for payload embedding are defined as the embeddable pixels in Algorithm 2 and 4. So, without loss of generality, we omit the embedding and extraction of *F* and *T* in the algorithms assuming that this pre-processing can be employed later for a practical application scenario.

**Algorithm 4**. extraction (*I*_*em*_, *F*, *T*)

**Input(s):** *I*_*em*_, *F*, *T*

**Output(s):** *D*, *I*

**Begin**

1 **Initialize:**

 *D* ← *Null*            ⊳ *D* is an empty array to store *data*

2 *I* ← downsample(*I*_*em*_, *F*)          ⊳ down-sample *I*_*em*_ by *F*

3 *C* ← interp(*I*, *F*)          ⊳ up-sampling *I* to *C* by factor *F*

4 {*y*′} ← *I*_*em*_ − *I*       ⊳ {*y*′} are the set of embedded pixels in *I*_*em*_

5 {*y*}←*C* − *I*         ⊳ {*y*} are the set of embeddable pixels in *C*

6 **for all** *y*′ and *y* **do**

7  *n* ← bitlength(*y*′)       ⊳ returns bit-length of *y*′ using Eq (8)

8  **if** *n* ≤ 1 + *T* **then**

9   *n*_*em*_ ← 2        ⊳ *n*_*em*_ is the number of embeddable bits

10  **else**

11   *n*_*em*_ ← *n* − *T*

12  **end if**

13  *p* ← getLSB(*y*, *n*_*em*_)           ⊳ returns *n*_*em*_-LSBs of *y*

14  *b* ← getLSB(*y*′, *n*_*em*_)          ⊳ returns *n*_*em*_-LSBs of *y*′

15  $\bar{b}\leftarrow \text{complement}\left(b\right)$     ⊳ returns 2’s complement of *b*

16  *X*_*b*_ ← bitXOR(*p*, *b*)          ⊳ a bit-wise XOR of *p* and *b*

17  $X_{\bar{b}}\leftarrow \text{bitXOR}\left(p,\bar{b}\right)$       ⊳ a bit-wise XOR of *p* and $\bar{b}$

18  **if** $\left|X_{b}|\leq |X_{\bar{b}}\right|$ **then**

19   *d* ← *b*            ⊳ data-bits are embedded in original

20  **else**

21   $d\leftarrow \bar{b}$        ⊳ data bits-are embedded in complement

22  **end if**

23  *D* ← append(*D*, *b*)    ⊳ append selected data bits, b with extracted Data, D

24 **end for**

25 Return *I*, *D*

**End**

### Proposed payload extraction with image recovery

Once the embedded image is sent to the receiver as showed in Fig 1, extraction of the embedded payload bits and recovery of the original image takes place. The data extraction with image recovery of our IRDH scheme is modeled in Algorithm 4. The original image, *I* can be restored instantly by down-sampling the embedded image, *I*_*em*_ (*i.e*., discarding the embedded pixels) and the embedded payload can be blindly extracted from the *I*_*em*_. Once the original image, *I* is restored, the interpolated image, *C* is reconstructed using interp(⋅). Now, for all embedded pixels *y*′ ∈ *I*_*em*_ − *I*, the bit length, *n* of *y*′ and the number of embedded bits, *n*_*em*_ in *y*′ are calculated using bitlength(⋅) and getnem(⋅) as per Eqs (8) and (6), respectively. Now, *n*_*em*_-number of LSBs of *y*′ ∈ *I*_*em*_ − *I* and corresponding *y* ∈ *C* − *I* are extracted as *b* and *p*, respectively using getLSB(⋅). Then *b* and *p* are compared using a bit-wise XOR operation to determine if the set of embedded bits is equal to *b* or its complement, $\bar{b}$ as per Eq (9). These operations are stated in Steps 13-22 of Algorithm 4. Therefore, all the extracted payload-bits, *d* are concatenated by append(⋅) to reconstruct the payload, *D* (which should be the same as the embedded payload, *data*).

$$\begin{array}{c} n=\{\begin{array}{cc} \;\lceil \log_{2}(y^{\prime })\rceil & \;\text{if}\;y^{\prime }>1\\ \;1 & \;\text{otherwise}\; \end{array} \end{array}$$ (8)

$$\begin{array}{c} d=\{\begin{array}{cc} \;b & \;\text{if}\;|X_{b}\left|\leq \right|X_{\bar{b}}|\\ \;\bar{b} & \;\text{otherwise} \end{array} \end{array}$$ (9)

### A working example

**Fig 4:**
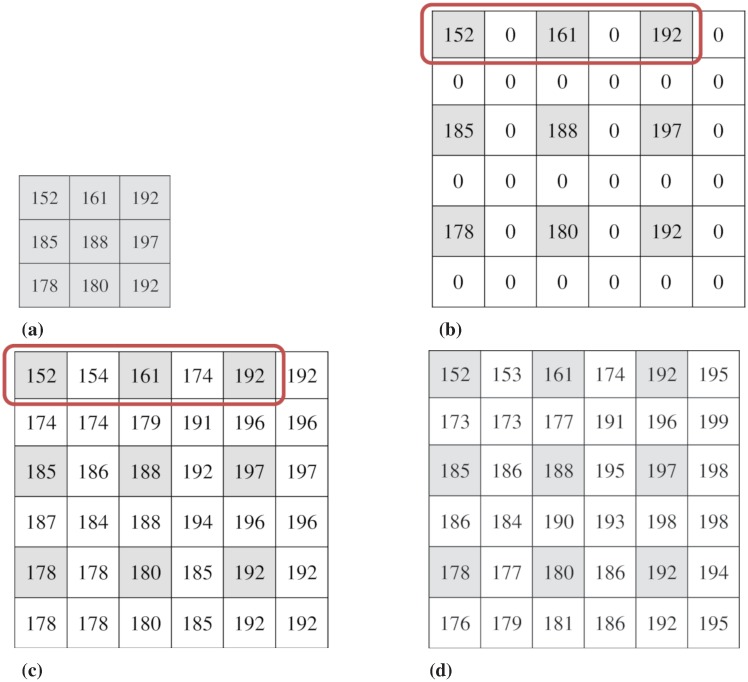
A minimal example of the proposed interpolation and embedding processes: (a) input image, (b) initial up-sampled image, (c) interpolated image, and (d) embedded image (the darker cells represent the original pixels).

We now briefly explain a minimal working example for our proposed IRDH scheme. As illustrated in Fig 4, an input image of size 3 × 3 is up-sampled to an image of size 6 × 6 using our SPI technique with *F* = 2 (see Algorithm 1). The up-sampling process is initialized with the interleaving zero columns and zero rows in the input image as in Fig 4(b). The darker pixels in the figure represent the original pixels and these pixels are kept unchanged. A directional block of five pixels is considered in all possible horizontal, vertical and diagonal directions to compute two unknown pixels from three known pixels as mentioned in Section Image up-sampling.

For a minimal working example, we explain here the first horizontal block, where the three known pixels are *y*_1_ = 152, *y*_3_ = 161 and *y*_5_ = 192 and the unknown pixels are *y*_2_ and *y*_4_. We initialize the up-sampling process for the unknown pixels, *y*_2_ and *y*_4_ with zeros, which are to be replaced by the respective interpolated values. We derive the set of three parabolic equations as in Eq (1) for the known three pixels, and solve those equations to obtain the coefficients, *a* = 2.75, *b* = −6.5 and *c* = 155.75. With the values of the coefficients, we now compute the unknown pixel values using Eq (2), *i.e*., *y*_2_ = 153.75 = 154 and *y*_4_ = 173.75 = 174, where the values are rounded up to the nearest integer numbers. This process of obtaining two interpolated pixels from three known pixels continues for all the directional blocks to obtain the final interpolated image with necessary padding as illustrated in Fig 4(c).

**Fig 5:**
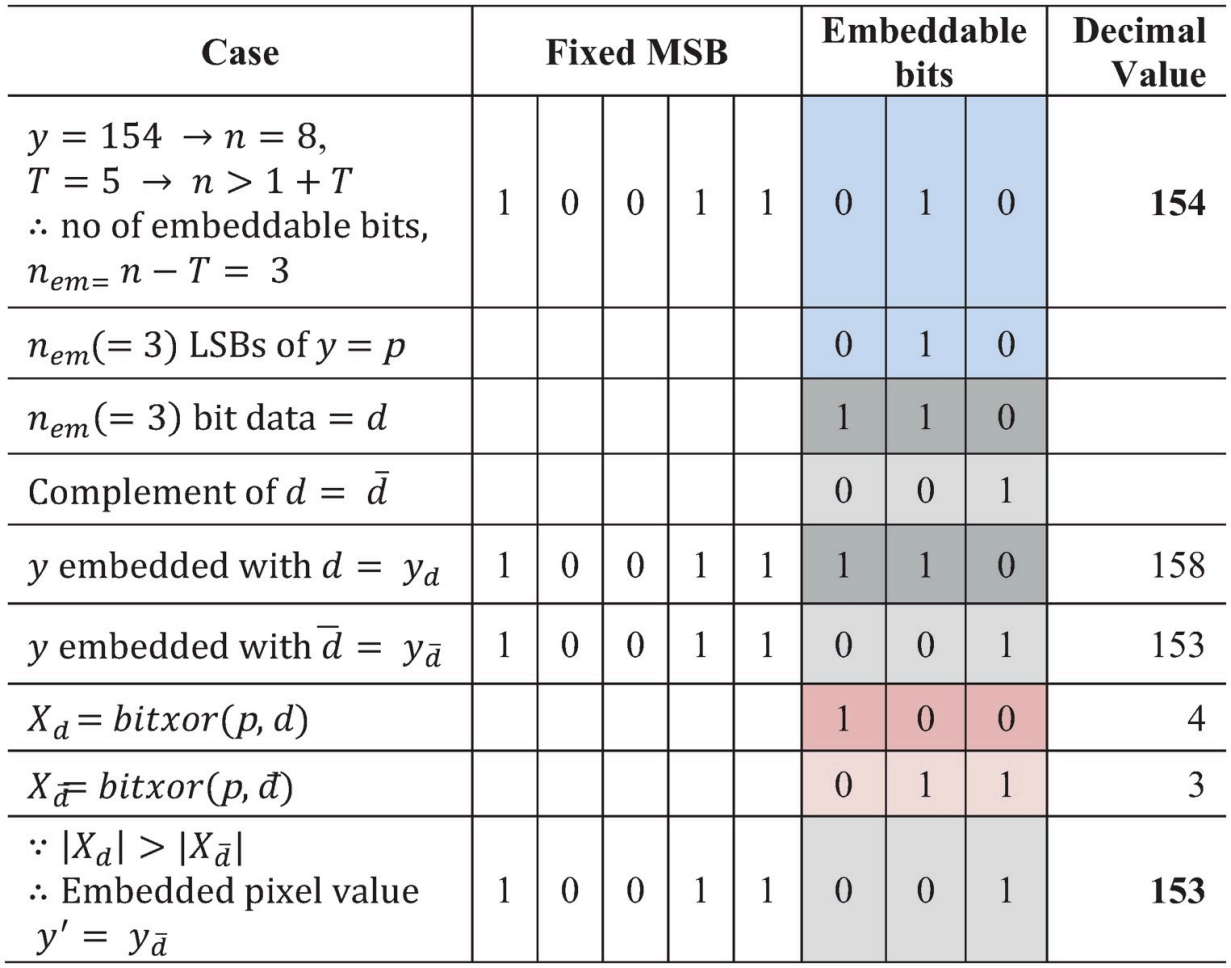
An example of proposed embedding in an embeddable pixel.

We now illustrate the process of our proposed adaptive embedding. The payload, a pseudo-random binary data (*e.g*., 1101100111011010011110111110101000011010001 …) is embedded into the embeddable pixels (white colored cells in the figure) and the embedded image is presented in Fig 4(d). The embedding process, for example, is now illustrated with *T* = 5 in Fig 5.

Let us consider the first embeddable pixel, *y* = (154)_10_ = (10011010)_2_. The bit-length of *y* is *n* = 8, thus from Eq 6, we get the number of embeddable bits, *n*_*em*_ = *n* − *T* = (8-5) = 3. The set of the three embeddable LSBs of *y* is *p* = 010. On the other hand, 3 bit of payload, *d* = 110. This set of payload bits, *d* is embedded in the embeddable LSBs of *y*, either in its original form (*i.e*. *d* = 110) or in complement form (*i.e*. $\bar{d}=001$). Now, the two versions of an embedded pixel, *y*_*d*_ and $y_{\bar{d}}$ can be obtained by substituting *d* or $\bar{d}$ respectively, for the set of embeddable LSBs, *p*. To select the final embedded pixel, we utilize a bit-wise correlation of *p* and *d*. We then compute the value, *X*_*d*_ = 4 using bit-wise XOR operation of *p* and *d*, and $X_{\bar{d}}=3$ using bit-wise XOR of *p* and $\bar{d}$. According to Eq 7, since $|X_{d}|>|X_{\bar{d}}$, replacement of the LSBs, *p* = 010 with the complement of data bits, $\bar{d}=001$ (*i.e*., $y_{\bar{d}}$ version of the embedded pixel) would cause minimum embedding distortion to the embeddable pixel, *y*. So, we obtain the final embedded pixel, $y^{\prime }=y_{\bar{d}}=(10011001{)}_{2}=(153{)}_{10}$.

On the other hand, in extraction, the original image can be exactly recovered by simply discarding the embedded pixels from the embedded image. Applying the reverse process of embedding, we can also completely extract the embedded payload from the embedded pixels as explained in Section Proposed payload extraction with image recovery.

## Results and analysis

This section presents the experimental results and analysis of our proposed IRDH scheme. In Section Computational efficiency of our SPI technique, we analyze the computational efficiency of our SPI technique, and in Section Rate-distortion performance of the proposed scheme, the embedding rate-distortion performance of our IRDH scheme is analyzed and validated by comparing it with some prominent IRDH schemes [13, 23, 25] and also with our previous scheme [15]. The experiment is carried out for a set of standard USC-SIPI grayscale test-images [40] of size 256 × 256. As mentioned in Section Image up-sampling, we have used a factor, *F* = 2, for up-sampling the original test images to a size of 512 × 512. The visual quality of the interpolated and embedded images are evaluated in terms of PSNR (peak signal to noise ratio) in dB and SSIM (structural similarity), two popular image quality metrics. Embedding rate and capacity are expressed in terms of bpp (bit per pixel) and the total number of embedded bits respectively. MATLAB R2016b with a 1.3 GHz Intel Core i5 CPU of 4 GB memory is used for all necessary implementations.

The PSNR values for the embedded images are computed in terms of mean square error (MSE) as given in Eqs (10) and (11). Here, *y*_*i*,*j*_ and $y_{i,j}^{\prime }$ are the pixel values of position (*i*, *j*) in an interpolated image and its embedded version, both of size *FM* × *FN*, respectively. Besides, SSIM values are computed using Eq (12), where *μ*_*y*_ and *μ*_*y*′_ are the average values of *y*_*i*,*j*_ and $y_{i,j}^{\prime }$, and $\sigma_{y}^{2}$ and $\sigma_{y^{\prime }}^{2}$ are the variance of *y*_*i*,*j*_ and $y_{i,j}^{\prime }$, respectively;*σ*_*y*,*y*′_ is the covariance of *y*_*i*,*j*_ and $y_{i,j}^{\prime }$; and *c*_1_ = (*k*_1_*L*)^2^ and *c*_2_ = (*k*_2_*L*)^2^ are two regularization constants for the 2^*L*^ − 1 dynamic range of the pixel values and a set of small constants, *k*_1_ and *k*_2_ [41]. Here, *L* is the bit-depth of the images.

$$\begin{array}{c} \text{MSE}=\frac{\sum_{j=1}^{FN}\sum_{i=1}^{FM}{\left(y_{i,j}-y_{i,j}^{\prime }\right)}^{2}}{FM\times FN} \end{array}$$ (10)

$$\begin{array}{c} \text{PSNR}=10\log\frac{{\left(2^{L}-1\right)}^{2}}{\text{MSE}} \end{array}$$ (11)

$$\begin{array}{c} \text{SSIM}=\frac{\left(2\mu_{y}\mu_{y^{\prime }}+c_{1}\right)\left(2\sigma_{y,y^{\prime }}+c_{2}\right)}{\left(\mu_{y}^{2}+\mu_{y^{\prime }}^{2}+c_{1}\right)\left(\sigma_{y}^{2}+\sigma_{y^{\prime }}^{2}+c_{2}\right)} \end{array}$$ (12)

### Computational efficiency of our SPI technique

The run-time and efficiency of our SPI technique are compared with Zhang *et al*.’s [13] PI technique in Table 2 in terms of their up-sampling run-time. As demonstrated in the table, for different test images, the average PSNR for the interpolated images with Zhang *et al*.’s [13] PI is 28.51 dB and that with our SPI is 27.17 dB. At the same time, the average run-time for our SPI is 1.81 sec, while that for Zhang *et al*.’s PI [13] is 10.68 sec. This means that, although the PSNR values are slightly reduced (4.7% on average) with SPI compared to PI, the SPI takes significantly less computational time (83% less on average). Thus computational complexity is significantly reduced with our SPI technique. Thereby, the overall embedding time of the proposed scheme is also significantly reduced. While the PSNR values for the interpolated images are slightly lower, the embedded image quality obtained with our proposed IRDH scheme eventually outperform Zhang *et al*.’s scheme, which is analyzed in the following sub-section. We here also note that, if there is no requirement of computational efficiency, Zhang *et al*.’s PI technique may be used for computing up-sampled images in our IRDH scheme. This would also improve the embedded image quality of our scheme further.

**Table 2:** Performance of the PI techniques.

Test Image	PSNR (dB)		Run-time (sec)		
	PI [13]	SPI (ours)	PI [13]	SPI (ours)	Efficiency (%)
Airfield	26.31	26.12	11.14	1.89	83.06
Baboon	23.03	21.89	10.04	1.79	82.19
Barbara	24.94	23.58	10.44	1.82	82.56
Boat	30.64	28.43	10.42	1.88	82.00
Bridge	25.78	24.86	10.48	1.82	82.63
Couple	29.16	26.51	10.20	1.79	82.49
Elaine	32.12	31.03	10.26	1.81	82.40
Goldhill	30.69	29.73	10.35	1.76	82.99
Lena	33.92	32.29	12.04	1.77	85.31
**Average**	**28.51**	**27.16**	**10.68**	**1.81**	**83.07**

### Rate-distortion performance of the proposed scheme

**Fig 6:**
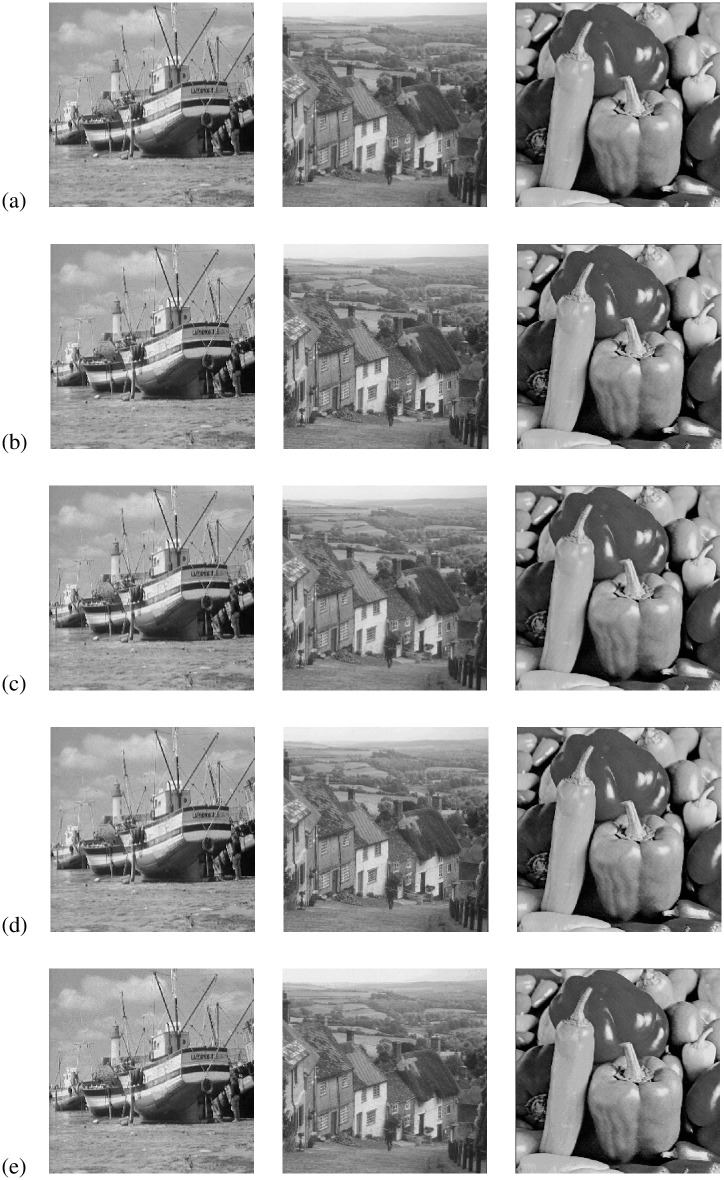
Example of output images: (a) interpolated images, (b) embedded images for *T* = 6, (c) embedded images for *T* = 5, (d) embedded images for *T* = 4 and (e) embedded images for *T* = 3. (Images in each row, from left: Boat, Goldhill and Peppers).

We observe that the embedded images by our scheme remain visually imperceptible compared to the interpolated images. Fig 6 illustrates the visual quality of interpolated and embedded versions of Boat, Goldhill and Lena images for different values of the capacity controlling parameter, *T*. Although this comparison is shown here only for a few sample test images, similar visual quality is also obtained for the other test images. A quantitative illustration of the rate-distortion performance is presented in Tables 3 and 4 in terms of PSNR (in dB), SSIM and embedding capacity (in total bits and bpp).

**Table 3:** Performance comparison of the proposed scheme.

Test Images	Performance Metric	Jung & Yoo [23]	Lee & Huang [25]	Zhang *et al*. [13]	Embedding with flag [15]				Embedding without flag (proposed)			
					T = 3	T = 4	T = 5	T = 6	T = 3	T = 4	T = 5	T = 6
Airfield	Total bits	529095	511793	698902	763301	567824	378693	215178	959807	763816	571255	393217
	bpp	2.018	1.953	2.666	2.912	2.166	1.445	0.821	3.661	2.914	2.179	1.500
	PSNR (dB)	23.85	23.76	22.53	27.61	33.50	39.55	45.42	33.46	39.53	45.67	50.92
	SSIM	0.8042	0.7970	0.8530	0.6494	0.8124	0.9140	0.9707	0.8120	0.9131	0.9691	0.9920
Baboon	Total bits	624709	637491	639317	676493	482541	311718	291703	872940	677741	494730	393217
	bpp	2.383	2.432	2.439	2.581	1.841	1.189	1.113	3.330	2.585	1.887	1.500
	PSNR (dB)	21.13	21.24	22.02	29.24	35.27	41.26	45.43	35.20	41.26	47.19	47.70
	SSIM	0.6549	0.6510	0.8399	0.7825	0.9093	0.9713	0.9890	0.9088	0.9707	0.9922	0.9955
Barbara	Total bits	494284	473225	590112	623713	437551	307388	318270	820322	628937	468163	393217
	bpp	1.886	1.806	2.251	2.379	1.669	1.173	1.214	3.129	2.399	1.786	1.500
	PSNR (dB)	23.30	23.59	24.10	30.19	36.23	42.02	45.44	36.18	42.22	47.27	46.47
	SSIM	0.7562	0.7570	0.8614	0.6582	0.8025	0.9012	0.9425	0.8047	0.9087	0.9600	0.9658
Boats	Total bits	455617	444284	440769	687627	509241	356615	260458	881426	695334	525975	393217
	bpp	1.738	1.695	1.681	2.623	1.943	1.360	0.994	3.362	2.652	2.006	1.500
	PSNR (dB)	26.60	26.59	29.15	28.68	34.59	40.46	45.43	34.53	40.52	45.96	48.05
	SSIM	0.7840	0.7870	0.9493	0.5738	0.7674	0.8976	0.9604	0.7696	0.9009	0.9615	0.9793
Bridge	Total bits	590842	568452	733636	618802	435031	301438	323632	813294	624163	462801	393217
	bpp	2.254	2.169	2.799	2.361	1.660	1.150	1.235	3.102	2.381	1.765	1.500
	PSNR (dB)	23.57	23.69	22.23	30.31	36.40	42.14	45.39	36.35	42.39	47.42	46.43
	SSIM	0.7204	0.7180	0.8479	0.8362	0.9345	0.9755	0.9873	0.9361	0.9787	0.9924	0.9938

**Table 4:** Performance comparison of the proposed scheme (contd.).

Test Images	Performance Metric	Jung & Yoo [23]	Lee & Huang [25]	Zhang *et al*. [13]	Embedding with flag [15]				Embedding without flag (proposed)			
					T = 3	T = 4	T = 5	T = 6	T = 3	T = 4	T = 5	T = 6
Couple	Total bits	423485	407754	586075	667161	473734	305315	299875	861865	667800	486558	393217
	bpp	1.615	1.556	2.236	2.545	1.807	1.165	1.144	3.288	2.547	1.856	1.500
	PSNR (dB)	25.59	25.48	27.43	29.58	35.59	41.52	45.44	35.51	41.53	47.45	47.56
	SSIM	0.8224	0.8200	0.9230	0.5986	0.7858	0.9081	0.9536	0.7838	0.9069	0.9690	0.9788
Elaine	Total bits	432844	415308	546098	688285	491972	318981	282726	884891	688432	503707	393217
	bpp	1.651	1.585	2.083	2.626	1.877	1.217	1.079	3.376	2.626	1.921	1.500
	PSNR (dB)	29.78	29.86	30.78	28.92	35.04	41.02	45.42	34.97	41.04	47.03	48.00
	SSIM	0.7327	0.7470	0.9255	0.5516	0.7640	0.9040	0.9628	0.7621	0.9032	0.9679	0.9864
Goldhill	Total bits	447244	438737	564789	613016	422962	288759	333906	809625	616294	452527	393217
	bpp	1.706	1.674	2.155	2.339	1.614	1.102	1.274	3.088	2.351	1.726	1.500
	PSNR (dB)	28.32	28.38	29.20	30.63	36.84	42.54	45.42	36.79	42.80	47.87	46.25
	SSIM	0.7974	0.7970	0.9285	0.6995	0.8454	0.9229	0.9573	0.8458	0.9269	0.9664	0.9795
Lena	Total bits	396268	380774	470653	656093	460611	326825	294693	852696	656654	491740	393217
	bpp	1.512	1.453	1.795	2.503	1.757	1.247	1.124	3.253	2.505	1.876	1.500
	PSNR (dB)	29.65	29.61	31.52	29.43	35.38	41.36	45.44	35.31	41.52	46.88	47.13
	SSIM	0.8660	0.8670	0.9546	0.5064	0.6818	0.8314	0.9207	0.6799	0.8327	0.9352	0.9678

As illustrated in Tables 3 and 4, our proposed scheme offers a relatively better-embedded image quality (*i.e*., the higher values of PSNR and SSIM) that gradually increases with the lower embedding rates and higher values of *T*. This is because, as mentioned in Section Proposed adaptive embedding, number of embeddable bits decreases (and respective embedded image quality improves), while *T* increases. Thus, a trade-off between the image quality and embedding rate is made with the adaptively chosen value of *T*, as explained in Algorithm 3, to obtain the best possible image quality for a given capacity requirement. Moreover, for similar embedding rates with a suitable value of *T*, our scheme is also observed to generate a better quality embedded image compared to that obtained by the schemes in [13, 15, 23, 25].

**Fig 7:**
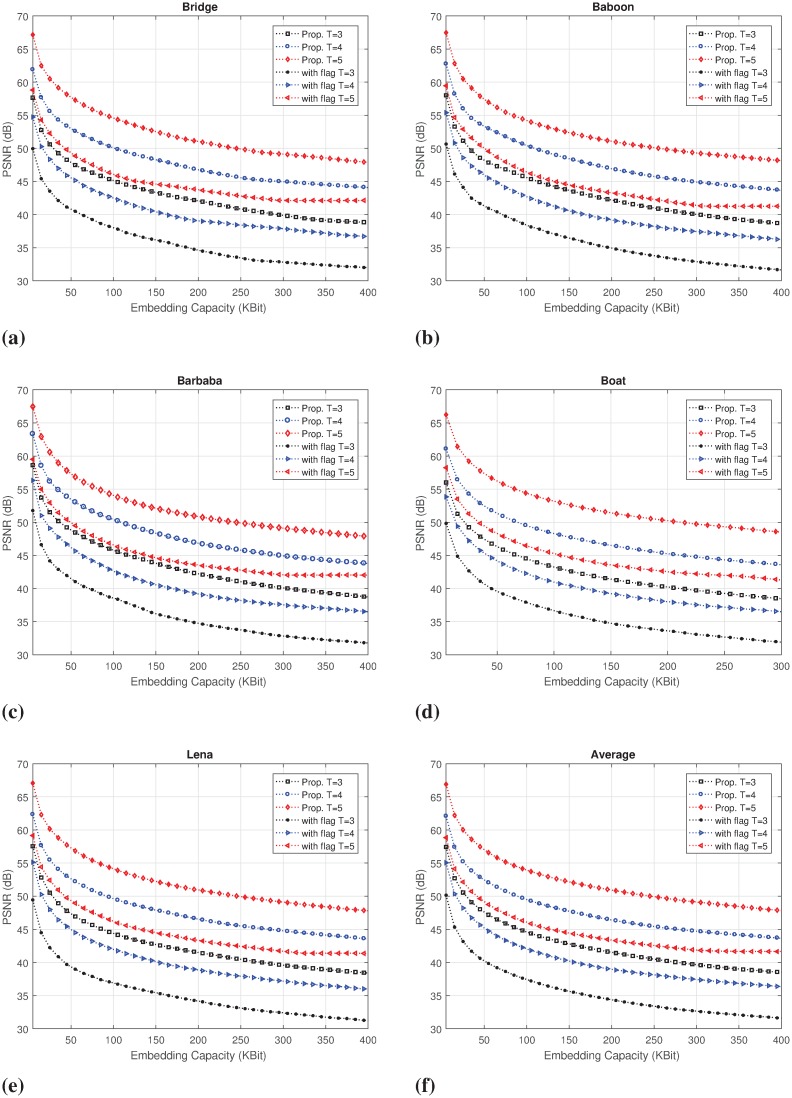
Embedding rate-distortion performance comparison of the proposed (without flag) scheme with our previous scheme (with flag) [15] for different values of *T*: (a) Bridge, (b) Baboon, (c) Barbara, (d) Boat, (e) Lena and (f) the average of all test-images.

**Fig 8:**
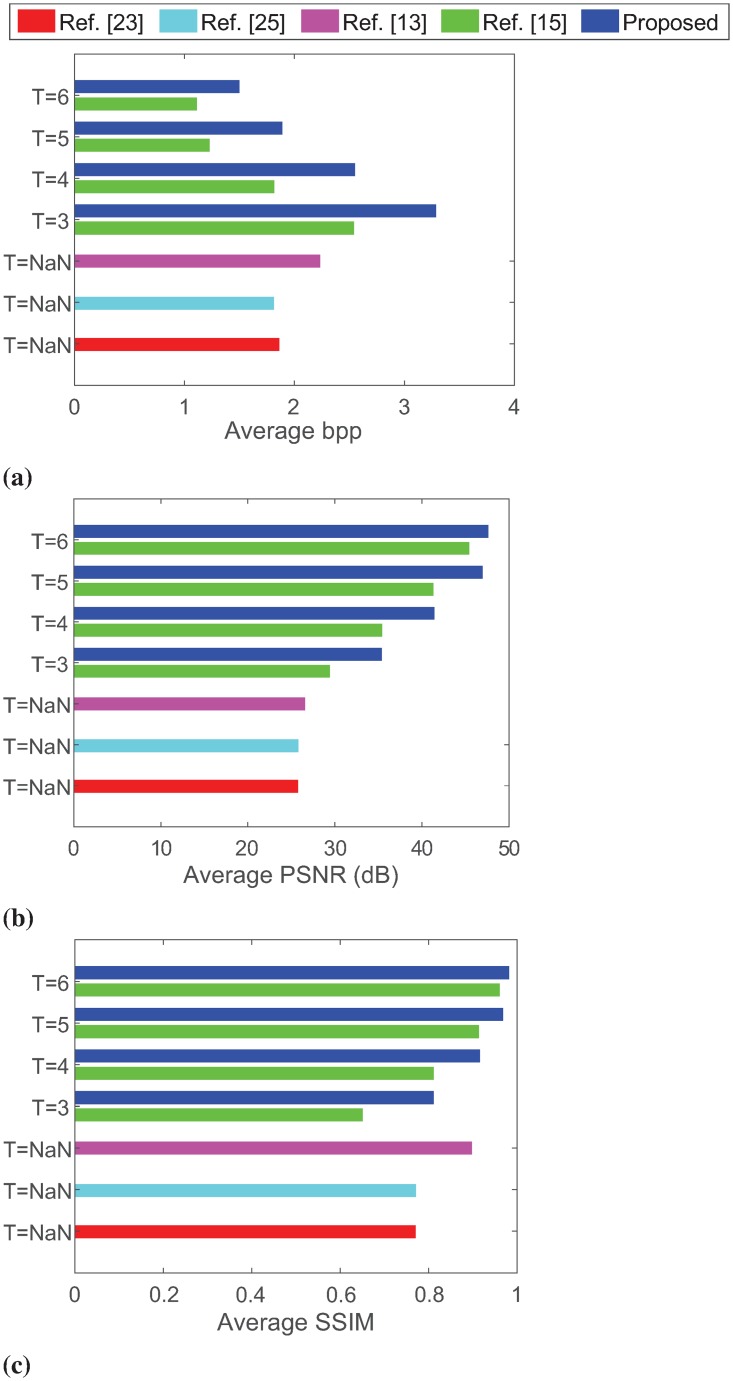
The average performance comparison of the proposed scheme with other schemes for different values of *T* in terms of (a) bpp, (b) PSNR, and (c) SSIM.

Particularly, unlike the schemes in [14] and [15], our scheme is modeled in Section Modeling our proposed IRDH scheme to avoid the use of flag bit. One more payload bit is rather embedded replacing the flag-bit in each embeddable pixel. This improvement of embedding rate and capacity with a better or almost similar embedded image quality becomes evident, while it is compared to [15] for the same values of *T* as in Figs 7 and 8. For example, with *T* = 4, our previous scheme [15] can embed up to 460611 bit payload with 1.76 bbp in Lena image, while the PSNR is 35.38 dB and SSIM is 0.6818; whereas, 656654 bit payload (2.51 bpp) with a better image quality (*i.e*., PSNR of 41.52 dB and SSIM of 0.8327) are embedded in our proposed scheme for the same value of *T*. This means that for a given value of *T*, our scheme achieves a higher embedding rate with improved image quality compared to the scheme in [15].

For the higher embedding capacity requirement (*i.e*., lower values of *T*), our proposed scheme also demonstrates a better rate-distortion performance than the scheme in [15]. For instance, with *T* = 5 and Lena image, our proposed scheme embeds total 491740 bits with 1.88 bpp, PSNR of 46.88 dB and SSIM of 0.9352. In contrast, with *T* = 4 and Lena image, the scheme in [15] can embed total 460611 bits of payload with 1.78 bpp, PSNR of 35.38 dB and SSIM of 0.6818, which are lower than the proposed scheme. This trend of improvement is also evident for the other test images in Fig 7.

Statistically, the scheme in [15] achieves an average maximum capacity ranging from 291 Kb to 666 Kb, PSNR from 45.43 dB to 29.40 dB and SSIM from 0.96 to 0.65 for *T* = [3, 6]. In contrast, the average embedding capacity of our proposed scheme varies in the dynamic range from 393 Kb to 861 Kb payload (*i.e*., 1.50 bpp to 3.29 bpp) for the same range of the capacity control parameter, with PSNR and SSIM values ranging from 35.37 dB to 47.61 dB and 0.811 to 0.982, respectively. With *T* = 5, for example, this improvement is recorded with 53.9% higher embedding capacity, 13.67% higher PSNR, and 5.93% higher SSIM, which are illustrated for the all considered values of *T* in Fig 8. This suggests that our proposed scheme offers both the higher dynamic range of embedding capacity and better-embedded image quality for a given capacity requirement.

Nevertheless, our proposed scheme also outperforms the prominent IRDH schemes in [13, 23, 25] for the individual test images in Tables 3 and 4. These improvements become more apparent in the average performance given in Table 5 and Fig 8. For example, Table 5 demonstrates that Zhang *et al*.’s scheme [13] offers the best embedding rate-distortion performance among the schemes in [13, 23, 25], which can embed an average size payload of 585 Kb (or embedding rate of 2.234 bpp) with PSNR of 26.55 dB and SSIM of 0.898. For our proposed scheme, considering its closer embedding rate to that of Zhang *et al*.’s scheme, we see that an embedding rate of 2.551 bpp (total 668 Kb) is obtained with a better PSNR of 41.42 dB and SSIM of 0.9158 for *T* = 4. Thus with *T* = 4, our proposed scheme offers 14% higher embedding rate with 56% and 2% better PSNR and SSIM, respectively. Moreover, we also note that the requirement of either a higher capacity or a better image quality can be attained with a lower or higher value of *T*, respectively for our proposed IRDH scheme.

**Table 5:** Comparison of average rate-distortion performance.

Schemes		Capacity/rate		Visual Quality	
		Total bits	bpp	PSNR (dB)	SSIM
Jung & Yoo [23]		488265	1.863	25.755	0.7709
Lee & Huang [25]		475313	1.814	25.80	0.7712
Zhang *et al*. [13]		585595	2.234	26.55	0.8981
Ours [15] (embedding with flag)	T = 3	666055	2.541	29.40	0.6507
	T = 4	475719	1.815	35.43	0.8115
	T = 5	321748	1.227	41.32	0.9140
	T = 6	291160	1.111	45.43	0.9605
Ours—proposed (embedding without flag)	T = 3	861874	3.288	35.37	0.8114
	T = 4	668797	2.551	41.42	0.9158
	T = 5	495273	1.889	46.97	0.9682
	T = 6	393217	1.500	47.61	0.9821

In summary, considering the computational efficiency and rate-distortion performance, our proposed IRDH scheme outperforms the existing IRDH schemes [13, 15, 23, 25]. For example, compared to our earlier scheme [15] (which is also better than the other three schemes, see Fig 8), the embedding capacity improvements are recorded 29%, 40%, 53% and 35% higher with 20%, 16.9%, 13.6% and 4.8% higher PSNR and 24.7%, 12.9%, 5.9% and 2.24% higher SSIM for *T* = 3, 4, 5 and 6, respectively. Thereby, the proposed scheme has demonstrated that it can effectively embed varying size payloads with the higher embedding rate and better image quality using a suitable capacity control parameter.

## Conclusion

A new adaptive IRDH scheme is presented and its computational efficiency and rate-distortion performance are analyzed in this paper for varying size of payloads. We have developed the SPI technique and utilized it for computing interpolated pixels. We have defined the capacity condition for adaptive embedding and formulated a capacity control parameter to attain that condition. Embedding process is modeled to utilize the logical correlation between the embeddable pixel and estimated versions of an embedded pixel to increase the embedding capacity. Extraction is modeled to blindly extract the embedded payload and to completely recover the original image, where these two processes were kept mutually independent to ensure a better user control in an application scenario.

Experimental results have substantiated that our proposed scheme can effectively embed varying size payload with a significantly higher embedding rate and better image quality compared to the prominent IRDH schemes. Embedding is carried out only in the interpolated pixels that would also minimize any possible concern of erratically modifying the input image and thus the proposed scheme could be useful in the military and medical image applications. With the adoption of suitable base conversion and compression techniques, the embedding capacity of the proposed IRDH scheme, however, may be further improved in the future.

## Supporting information

S1 File Sample MATLAB scripts. The given MATLAB scripts execute the proposed scheme with a set of given inputs, and write the statistical performance (in a Microsoft Excel file) and output images of the scheme.
(ZIP)
