# Supplementary figures and images for: High capacity reversible data hiding with interpolation and adaptive embedding
Source: PLoS One. 2019 Mar 6;14(3):e0212093. doi: 10.1371/journal.pone.0212093 (PMC6402661; doi:10.1371/journal.pone.0212093)

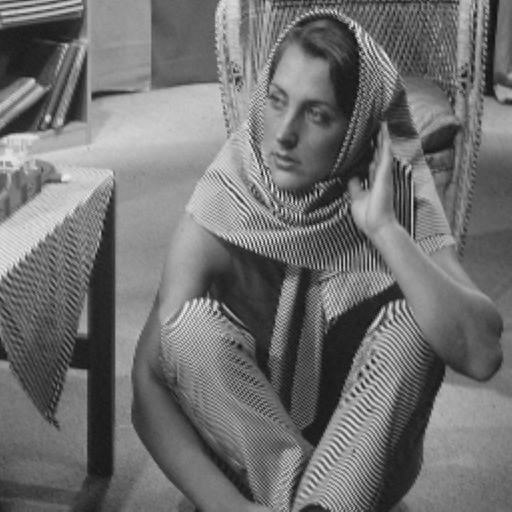

Supplement: S1 File — The given MATLAB scripts execute the proposed scheme with a set of given inputs, and write the statistical performance (in a Microsoft Excel file) and output images of the scheme. (ZIP) [file pone.0212093.s001.zip › Result/Barbara_quad.tif]

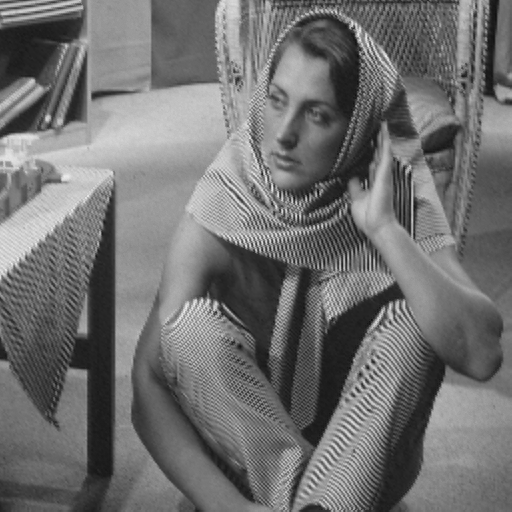

Supplement: S1 File — The given MATLAB scripts execute the proposed scheme with a set of given inputs, and write the statistical performance (in a Microsoft Excel file) and output images of the scheme. (ZIP) [file pone.0212093.s001.zip › Result/Barbara_quad_XOR_T=5.tif]

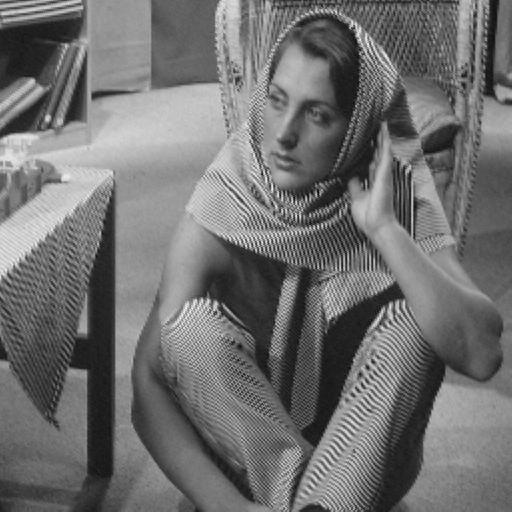

Supplement: S1 File — The given MATLAB scripts execute the proposed scheme with a set of given inputs, and write the statistical performance (in a Microsoft Excel file) and output images of the scheme. (ZIP) [file pone.0212093.s001.zip › Result/Barbara_quad_XOR_T=6.tif]

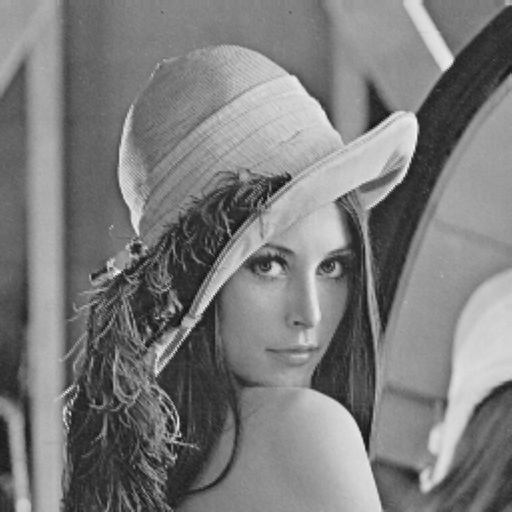

Supplement: S1 File — The given MATLAB scripts execute the proposed scheme with a set of given inputs, and write the statistical performance (in a Microsoft Excel file) and output images of the scheme. (ZIP) [file pone.0212093.s001.zip › Result/Lena_quad.tif]

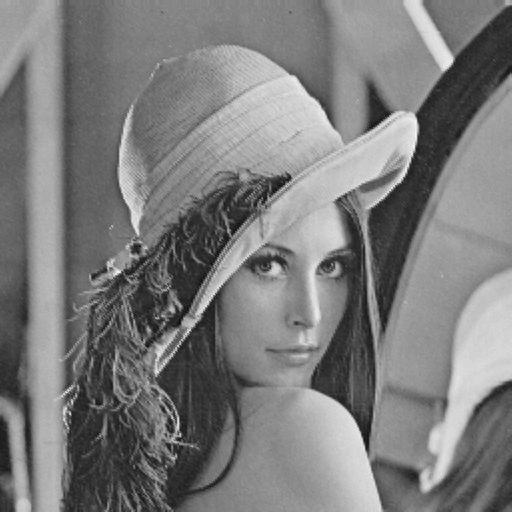

Supplement: S1 File — The given MATLAB scripts execute the proposed scheme with a set of given inputs, and write the statistical performance (in a Microsoft Excel file) and output images of the scheme. (ZIP) [file pone.0212093.s001.zip › Result/Lena_quad_XOR_T=5.tif]

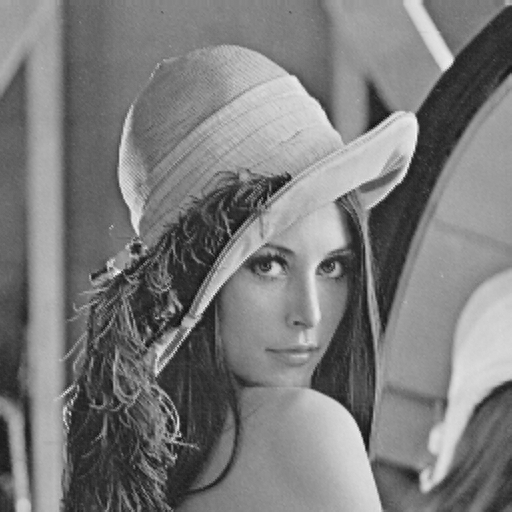

Supplement: S1 File — The given MATLAB scripts execute the proposed scheme with a set of given inputs, and write the statistical performance (in a Microsoft Excel file) and output images of the scheme. (ZIP) [file pone.0212093.s001.zip › Result/Lena_quad_XOR_T=6.tif]

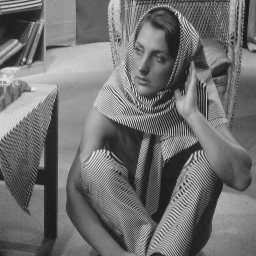

Supplement: S1 File — The given MATLAB scripts execute the proposed scheme with a set of given inputs, and write the statistical performance (in a Microsoft Excel file) and output images of the scheme. (ZIP) [file pone.0212093.s001.zip › Test-image/Barbara.tif]

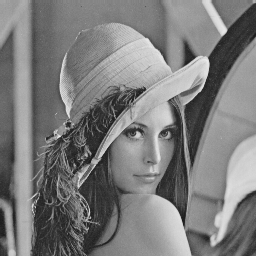

Supplement: S1 File — The given MATLAB scripts execute the proposed scheme with a set of given inputs, and write the statistical performance (in a Microsoft Excel file) and output images of the scheme. (ZIP) [file pone.0212093.s001.zip › Test-image/Lena.tif]
